# Journal Court: A Novel Approach to Incorporate Medicolegal Education into an Emergency Medicine Journal Club

**DOI:** 10.21980/J8093T

**Published:** 2025-01-31

**Authors:** Kevin McGurk, Mary Jordan, Bradley Davis

**Affiliations:** * Medical College of Wisconsin, Department of Emergency Medicine, Milwaukee, WI

## Abstract

**Audience:**

The target audience includes health professions students, residents, and fellows who participate in journal clubs.

**Introduction:**

Journal club plays an important role in teaching emergency medicine residents how to critically evaluate medical literature and apply it to their clinical practice. While there is some consensus on the general goals and objectives of journal club, significant variability exists between how different residency programs design and conduct them.^1^ Papers selected may address similar or disparate topics, highlight specific research applications, or demonstrate diverging evidence on a specific issue.^2^–^5^ While numerous approaches have been implemented and described, they do not traditionally entail a trial-based format.

More than 7% of practicing physicians have a malpractice claim annually and more than one third will be sued in their lifetime.^6^,^7^ Some estimates indicate 75% of emergency medicine physicians will be named in a medical malpractice suit during their career.^8^ Despite this, the American College of Graduate Medical Education (ACGME) has no specific requirement for medicolegal instruction during emergency medicine training.^9^ By structuring journal club to encompass a hypothetical medical malpractice lawsuit, our program sought to provide instruction on this topic while also fostering improved resident enthusiasm and participation.

**Educational Objectives:**

By the end of this exercise, participants should: 1) identify the four necessary elements for a malpractice claim, 2) understand the basic structure of medical malpractice litigation, and 3) critically analyze medical literature representing diverging viewpoints or conclusions.

**Educational Methods:**

Residents read two papers regarding fluid resuscitation in sepsis and a fictional case narrative and associated medical malpractice complaint. The case described a septic patient with a history of congestive heart failure who clinically decompensates after large volume IV fluid administration.^10^,^11^ After a brief faculty-led discussion on medical malpractice, a mock trial was conducted. Rather than a more conventional journal club format, the two presenting residents discussed the papers by citing them as evidence in their role as expert witnesses providing testimony on behalf of the plaintiff or defense. Each expert witness explained the strengths of their respective paper and highlighted the weaknesses of the opposing paper. A jury made up of resident attendees then deliberated and rendered a verdict followed by an open discussion among the entire group regarding both papers.

**Research Methods:**

At the conclusion of the journal club, residents in attendance were asked to complete a brief and anonymous survey evaluating the activity. Questions utilized a 5-point Likert scale to assess the journal club’s utility for teaching about research appraisal and the medical malpractice process.

**Results:**

Among respondents, 14/15 (93.3%) agreed or strongly agreed that the journal club had improved their understanding of clinical research and 14/15 (93.3%) agreed or strongly agreed that the journal club had improved their understanding of medical malpractice litigation. Narrative feedback was limited but uniformly positive and included comments such as “Great journal club” and “This was one of the most engaging journal clubs I have ever been to, love the content as well as the format.”

**Discussion:**

Journal club is a novel and effective venue for introducing medicolegal education into an emergency medicine residency curriculum. With a brief presentation and informal mock trial, residents were exposed to the elements of malpractice as well as pertinent state laws regarding medical malpractice. They were able to verbalize understanding of these legal tenets and effectively apply them to a simulated scenario. Additionally, they were able to effectively analyze and compare medical literature and understand implications for clinical practice.

The exercise required a little more preparation than the conventional journal club format but was inexpensive, well-received and could be easily replicated.

**Topics:**

Journal club, Emergency Medicine, medical malpractice, litigation, lawsuit, Torts, medical education, legal liability, medical errors.

## USER GUIDE

**Table:** 

List of Resources:	
Abstract	1
User Guide	3
Small Groups Learning Materials	6
Appendix 1: Journal Court Outline/Itinerary	6
Appendix 2: Sample Case Narrative and Malpractice Complaint	8
Appendix 3: Jury Summons	10
Appendix 4: Jury Instructions	11

**Learner Audience:**

Medical Students, Interns, Residents, Other Health Professions Students

**Time Required for Implementation:**

Journal articles were selected approximately one month in advance, but less lead time would likely be sufficient. The papers, case narrative, medical malpractice claim, and jury summons were sent one week prior to the event. The mock trial lasted for approximately one hour, but length could be adjusted to accommodate for varying case complexity and number of participants involved.

**Topics:**

Journal club, emergency medicine, medical malpractice, litigation, lawsuit, torts, medical education, legal liability, medical errors.

**Objectives:**

By the conclusion of the journal court exercise, participants should be able to:

1. Identify the four necessary elements for a malpractice claim
2. Understand the basic structure of medical malpractice litigation
3. Critically analyze medical literature representing diverging viewpoints or conclusions

### Linked objectives and methods

Prior studies have demonstrated the efficacy of mock trial and similar exercises as tools to facilitate medicolegal education. A simulated trial can serve as a model to teach critical thinking, and residents studied across multiple specialties have found the format engaging and helpful.^12^–^16^ Adult learning theory advocates for interactive teaching methodology with audience participation, and mock trial provides such a structure.^15^–^16^ Prior research has shown that simulated medical malpractice litigation can improve residents’ understanding of the consequences of medical errors, bolster confidence in entering a medical claim, and develop improved communication skills.^12^–^15^

For our journal court, faculty selected two papers offering differing perspectives on the approach to IV fluid resuscitation in septic patients with congestive heart failure or end stage renal disease.^10^,^11^ A fictional case narrative was then written by faculty during which a septic patient with heart failure suffers an adverse outcome following large volume IV fluid administration in the ED. A medical malpractice complaint was written to imitate conventional litigation documents. By framing the articles to be discussed around concrete applications involving clinical practice and litigation, the activity capitalized on core elements of adult learning theory.^17^,^18^ Both articles were provided to residents as were the case narrative and malpractice complaint. In the week prior to journal club, a fictional jury summons was sent to six residents who had indicated they would be in attendance. At the journal club, a brief faculty-led presentation was provided regarding the basic format of medical malpractice litigation and pertinent state-specific rules. The four requirements for a valid malpractice claim were explained including the presence of a physician-patient relationship, demonstrable patient harm, physician negligence, and negligence as the proximate cause to any injuries. These elements can be best remembered as the four D’s: duty, damages, dereliction, and direct cause (objectives 1 and 2).

An informal mock trial was then initiated. Though it lacked the solemnity of a trial (tacos and margaritas are rarely allowed in a courtroom), the same basic structure was followed. During the proceedings, the two residents responsible for both papers did not discuss them in our customary format. Instead, each played the role of an expert witness with one representing the plaintiff while the other represented the defense. In support of their testimony, each resident presented their respective paper and its strengths while also highlighting weaknesses or limitations of the opposing paper (objectives 2 and 3). Following their testimony, the resident jurors briefly deliberated before rendering a verdict at which time all those in attendance could discuss the papers in a less structured format.

### Recommended pre-reading for facilitator

- Gottlieb M, King A, Byyny R, Parsons M, Bailitz J. Journal Club in residency education: An evidence-based guide to best practices from the Council of Emergency Medicine Residency Directors. *West J Emerg Med*. 2018;19(4):746–755.
- Nepps ME. The basics of medical malpractice: a primer on navigating the system. *Chest*. 2008;134(5):1051–1055. doi:10.1378/chest.08-0186
- Gilbert WM, Fadjo DE, Bills DJ, Morrison FK, Sherman MP. Teaching malpractice litigation in a mock trial setting: a center for perinatal medicine and law. *Obstet Gynecol*. 2003;101(3):589–593. doi:10.1016/s0029-7844(02)03133-2
- Bono MJ, Wermuth HR, Hipskind JE. Medical Malpractice. In: *StatPearls*. Treasure Island (FL): StatPearls Publishing; October 31, 2022.

### Learner responsible content (LRC)

Attendees should read the two assigned articles addressing fluid resuscitation in sepsis and the fictional case narrative and malpractice complaint included in Appendix 2.^10^,^11^ The selected papers provide evidence on fluid administration in sepsis and offer background that allows for nuanced and potentially diverging positions on the appropriateness of large volume fluid use in septic patients with a history of congestive heart failure. Alternative papers could be substituted to address different clinical or research topics as desired.

Learners participating in the mock trial are recommended to read the summary of medical malpractice referenced to familiarize themselves with the basics of malpractice litigation.

- Bono MJ, Wermuth HR, Hipskind JE. Medical malpractice. In: *StatPearls*. Treasure Island (FL): StatPearls Publishing; October 31, 2022.

### Small group application exercise (sGAE)

Appendix 1: Journal Court Outline/Itinerary
Appendix 2: Sample Case Narrative and Malpractice Complaint
Appendix 3: Jury Summons
Appendix 4: Jury Instructions

### Materials List

For the journal club described, the faculty member provided dinner for attendees and purchased a novelty gavel, but no specific materials are required for the exercise. It can easily be conducted at little or no cost.

### Results and tips for successful implementation

Residents participated in spirited debate on the hypothetical case before the verdict was ultimately rendered. At the conclusion of journal court, they were asked to complete a brief and anonymous survey evaluating the activity. Questions utilized a 5-point Likert scale to assess the journal club’s utility for teaching about research appraisal and the medical malpractice process. Among attendees, 15/18 (83.3%) completed the survey with 14/15 (93.3%) respondents who agreed or strongly agreed that the journal club had improved their understanding of clinical research, and 14/15 (93.3%) who agreed or strongly agreed that the journal club had improved their understanding of medical malpractice litigation. Residents enjoyed the journal court format with 15/15 (100%) respondents agreeing or strongly agreeing with the statement, “I would like future journal club(s) to use a similar case/trial-based format.” Narrative feedback was limited but similarly positive with residents praising the event and offering opinions on the case results. Representative comments included, “Great journal club!” and “This was one of the most engaging journal clubs I have ever been to, love the content as well as the format.”

One impetus for this novel format was to improve resident engagement during journal club. Discussing articles that trainees were interested in was a necessary step to ensure robust participation and as such resident inclusion in the article selection process should be strongly encouraged. Faculty assistance is recommended for writing or editing the case narrative to ensure a viable scenario has been posed that allows for balanced debate on contrasting clinical positions. The case narrative and malpractice complaint can be shared with participants in advance (along with the assigned journal articles) to ensure efficiency, or they can be presented on the day of journal court to better replicate a trial scenario.

Empaneling a jury of residents was well-received and allowed for additional active roles during the exercise. Were a program to employ the same principles on a larger size and scale, more roles could easily be added including lawyers, a larger jury, or additional experts to provide testimony.

State malpractice laws can vary substantially. Journal Court organizers should review the basics applicable to their location to better ensure some degree of regional fidelity.

### Pearls

Four elements of a valid malpractice claim:

○ 1. presence of a physician-patient relationship
○ 2. demonstrable patient harm
○ 3. physician negligence
○ 4. negligence as the proximate cause to any injuries.

These elements can be best remembered as the **four D’s**: **duty, damages, dereliction, and direct cause.**

## SMALL GROUPS LEARNING MATERIALS

### Appendix 1 Journal Court Outline/Itinerary

Brief Outline and Materials for a Journal Club Medical Malpractice Trial:

**Agenda:** (Approximately one hour as outlined but easily adjustable)

#### A. Introduction (5 minutes)

- Overview of agenda
- Brief explanation of the four essential elements typically necessary to establish a medical malpractice claim:
  1. Duty: The healthcare provider owed a duty of care to the patient. This typically entails demonstrating that a doctor-patient relationship exists.
  2. Breach of Duty: The healthcare provider breached the standard of care by failing to act in a manner consistent with what a reasonable clinician would have done in similar circumstances.
  3. Causation: There must be a direct link between the healthcare provider’s breach of duty and the patient’s injuries. This means demonstrating that the physician’s actions (or lack thereof) directly caused the harm.
  4. Damages: The patient must have suffered actual harm, whether it’s physical, emotional, or financial, as the result of the healthcare provider’s breach of duty.

#### B. Case Summary/Opening Statements (5 minutes)

- Briefly review the facts of the case as outlined in the case narrative. Optional if using mock attorneys in addition to the expert witnesses.
  ○ Plaintiff’s Attorney briefly presents the case, introduces the parties, and outlines the alleged malpractice.
  ○ Defendant’s Attorney: Responds with a concise overview of the defense’s position.

#### C. Presentation of Plaintiff’s Case/Expert Testimony (15 minutes)

- The plaintiff’s expert witness offers testimony by reviewing the peer-reviewed paper assigned in support of their positions.
  ○ *Optional:* This can also occur as “direct examination of the witness” conducted by the mock attorneys if they are utilized.

#### D. Cross-Examination by the Defense (5 minutes)

- Opposing expert or attorney (if used) asks questions to highlight weaknesses and limitations regarding the paper presented.

#### E. Presentation of Defense’s Case/Expert Testimony (15 minutes)

- The defense’s expert witness offers testimony by reviewing the peer-reviewed paper assigned in support of their positions.
  ○ *Optional:* This can also occur as “direct examination of the witness” conducted by mock attorneys if they are utilized.

#### F. Cross-Examination by the Plaintiff (5 minutes)

- Opposing expert or attorney (if used) asks questions to highlight weaknesses and limitations regarding the paper presented.

#### G. Closing Arguments (5 minutes)

- Plaintiff’s Attorney: Summarizes the case and briefly requests compensation.
- Defendant’s Attorney: Presents a concise summary of the defense’s position and requests dismissal.

#### H. Jury Instructions (2 minutes) - *optional*

- The judge provides a very brief set of instructions to the jury. A sample script for jury instructions is provided in Appendix 4.

#### I. Jury Deliberations and Verdict (5 minutes)

- The jury quickly deliberates.
- A jury foreperson delivers the verdict.

#### J. Adjournment and Open Discussion with Journal Club Attendees

- Participants and attendees can discuss further as time or interest allows.

### Appendix 2 Sample Case Narrative and Malpractice Complaint

#### Brief Narrative of the Case

Mark Arnold, a 68-year-old male with past medical history of hypertension, diabetes mellitus, Hyperlipidemia, and congestive heart failure presented to the Big Wisconsin Hospital emergency room due to fever and weakness. Mr. Arnold had developed urinary urgency and discomfort that progressively worsened over approximately three days. On the morning of July 1, 2022, he felt feverish and weak, and his wife was concerned he might also be confused. In the emergency room, Mr. Arnold was cared for by Dr. Charles Watson, an emergency medicine physician at Big Wisconsin Hospital. Upon arrival, Mr. Arnold and his wife Susan explained his recent history and relayed relevant past medical history including diabetes and congestive heart failure. At the time of triage, his vital signs were as follows:

Heart Rate: 110. Respiratory rate: 20 Temperature: 101.3° F Blood Pressure: 86/56

Dr. Watson documented suspicion for sepsis secondary to a urinary tract infection and ordered tests including blood work, urine studies, a chest X ray, a CT scan of the head, a CT scan of the of the abdomen and pelvis, and cultures of both blood and urine. He ordered antipyretics, intravenous antibiotics (vancomycin and cefepime) and a 1L IV fluid bolus using 0.9% normal saline. Labs were notable for an elevated lactic acid level (4.5), a white blood cell count of 18,000, and a urinalysis that had > 100 WBCS and was nitrite positive. CT imaging of the abdomen and pelvis demonstrated abnormality along the right kidney suggesting pyelonephritis.

Following medication and 1L of IV fluids, Mr. Arnold’s blood pressure had minimally improved to 92/60 and his heart rate decreased to 95/minute. Given persistent hypotension, Dr. Watson administered additional IV fluids to reach a total volume of 30 mL/Kg and admitted Mr. Arnold to the medical intensive care unit.

Upon arrival to the ICU, Mr. Arnold complained of new and worsening shortness of breath. His respiratory rate had increased and oxygen saturation decreased. The ICU physician, Dr. Mitchell Holmes, documented increased work of breathing and decreased breath sounds bilaterally with jugular venous distention concerning for volume overload. Mr. Arnold was provided respiratory support via face mask but rapidly became sicker and subsequently needed to be intubated. His medical course was later complicated by prolonged mechanical ventilatory support and subsequent ventilator-associated pneumonia. Following more than a week in the ICU, he developed acute repiratory distress symptoms (ARDS) and had a cardiac arrest. While he was successfully resuscitated, he was later found to have sustained an anoxic brain injury with permanent neurocognitive deficits.

Mr. Arnold and his wife, represented by the law firm Lewis, Graham, Sell and Bollinger, have filed suit against the emergency physician Dr. Watson.

#### Malpractice Complaint

##### Mark Arnold vs Charles Watson, MD

1. That the Plaintiff is a resident of Milwaukee County, Wisconsin.
2. That Defendant Charles Watson, M.D. is a physician employed by Big Wisconsin Hospital providing emergency medical care and was acting within the scope of that employment relationship when he failed to follow the applicable standard of medical care during the treatment of the Plaintiff on or about July 1, 2022, which proximately resulted in a physical injury to the Plaintiff.
3. That these medical mistakes occurred on July 1, 2022, in the State of Wisconsin.
4. That on or about July 1, 2022, the Plaintiff came to the emergency room at Big Wisconsin Hospital due to fever and weakness. He was diagnosed with sepsis secondary to a kidney infection.
5. That following the Plaintiff’s lab work, he received a large volume of IV fluids (30 mL/kg) despite a known history of heart failure.
6. That the standard of medical care applicable to the Defendants after informed by the plaintiff and his wife of his medical history – including heart failure—was to avoid over administration of IV fluids.
7. As a direct result of the Defendants breaching the applicable standard of medical care owed to the Plaintiff by administering large volume IV fluids despite his heart failure, the Plaintiff suffered a physical injury to his body.
8. That as a direct and proximate result of the breach of the applicable standard of medical care by the Defendant, the Plaintiff has suffered harm. These harms include: 1) suffered conscious pain and suffering both in the past, and it is expected by her physicians, the future, 2) incurred medical expenses in the past and will incur future medical expenses, 3) suffered mental and emotional sorrow and anguish, 4) suffered permanent physical injuries (anoxic brain injury) and disfigurement, and 5) was required to undergo additional medical procedures and has sustained other damages.
9. That all of the injuries and damages sustained by the Plaintiff were the direct and proximate result of the negligent actions and breaches of the applicable standards of medical care by the Defendant without any act or omission on the part of the Plaintiff directly thereunto contributing.

### Appendix 3 Jury Summons

#### [Institutional Logo if so desired] Official Jury Summons

Greetings, you are hereby summoned for jury service and to appear in person before the court of the [Institution/Program] Journal Club.

Please report to Journal Court on [Date] at [address].

A person who fails to comply with this summons, or who knowingly provides false information in a request for an exemption or to be excused from jury service, is subject to a punishable contempt action.

Thank you in advance for your service.

-The Court

### Appendix 4 Jury Instructions

#### Jury Instructions*

In diagnosing and treating (the plaintiff’s) condition, (Doctor ***) was required to use the degree of care, skill, and judgment which reasonable physicians would exercise in the same or similar circumstances. A doctor who fails to conform to this standard is negligent. The burden is on the plaintiff to prove that (Doctor ***) was negligent.

A doctor is not negligent, however, for failing to use the highest degree of care, skill, and judgment or solely because a bad result may have followed his or her care and treatment. The standard you must apply in determining if (Doctor ***) was negligent is whether (he/she) failed to use the degree of care, skill, and judgment that a reasonable physician would exercise.

You have heard testimony during this trial from doctors who have testified as expert witnesses. The reason for this is because the degree of care, skill, and judgment that a reasonable doctor would exercise is not a matter within the common knowledge of laypersons. This standard is within the special knowledge of experts in the field of medicine and can only be established by the testimony of experts. You, therefore, may not speculate or guess what the standard of care, skill, and judgment is in deciding this case but rather must attempt to determine it from the expert testimony that you heard during this trial. In determining the weight to be given an opinion, you should consider the qualifications and credibility of the expert and whether reasons for the opinion are based on facts in the case. You are not bound by any expert’s opinion.

The cause question asks whether there was a causal connection between negligence on the part of (Doctor ***) and (plaintiff)’s condition. A person’s negligence is a cause of an injury if the negligence was a substantial factor in producing the present condition of the plaintiff’s health. This question does not ask about “the cause” but rather “a cause.” There can be more than one cause of an injury. Negligence can cause harm or it can be the result of the natural progression of the injury or condition. In addition, the injury can be caused jointly by a person’s negligence and also the natural progression of the condition.

If you conclude from the evidence that the present condition of (plaintiff)’s health was caused jointly by (Doctor ***)’s negligence and also the natural progression of illness, then you should find that the (Doctor ***)’s negligence was a cause of the (plaintiff)’s present condition of health.

This question asks you to determine whether the condition of (plaintiff)’s health, as it was when (plaintiff) placed (himself/herself) under the doctor’s care, has been aggravated or further impaired as the result of the negligence of (Doctor ***)’s diagnosis and treatment.
